# Neutrophil Extracellular Traps and Bacterial Biofilms in Middle Ear Effusion of Children with Recurrent Acute Otitis Media – A Potential Treatment Target

**DOI:** 10.1371/journal.pone.0053837

**Published:** 2013-02-05

**Authors:** Ruth B. Thornton, Selma P. Wiertsema, Lea-Ann S. Kirkham, Paul J. Rigby, Shyan Vijayasekaran, Harvey L. Coates, Peter C. Richmond

**Affiliations:** 1 School of Paediatrics and Child Health, The University of Western Australia, Perth, Australia; 2 Telethon Institute for Child Health Research, Centre for Child Health Research, University of Western Australia, Perth, Australia; 3 Centre for Microscopy, Characterisation and Analysis, The University of Western Australia, Perth, Australia; 4 Department of Otolaryngology, Head and Neck Surgery, Princess Margaret Hospital for Children, Perth, Australia; 5 Department of Otolaryngology, Head and Neck Surgery, The University of Western Australia, Perth, Australia; Universite de la Mediterranee, France

## Abstract

**Background:**

Bacteria persist within biofilms on the middle ear mucosa of children with recurrent and chronic otitis media however the mechanisms by which these develop remain to be elucidated. Biopsies can be difficult to obtain from children and their small size limits analysis.

**Methods:**

In this study we aimed to investigate biofilm presence in middle ear effusion (MEE) from children with recurrent acute otitis media (rAOM) and to determine if these may represent infectious reservoirs similarly to those on the mucosa. We examined this through culture, viability staining and fluorescent *in situ* hybridisation (FISH) to determine bacterial species present. Most MEEs had live bacteria present using viability staining (32/36) and all effusions had bacteria present using the universal FISH probe (26/26). Of these, 70% contained 2 or more otopathogenic species. Extensive DNA stranding was also present. This DNA was largely host derived, representing neutrophil extracellular traps (NETs) within which live bacteria in biofilm formations were present. When treated with the recombinant human deoxyribonuclease 1, Dornase alfa, these strands were observed to fragment.

**Conclusions:**

Bacterial biofilms, composed of multiple live otopathogenic species can be demonstrated in the MEEs of children with rAOM and that these contain extensive DNA stranding from NETs. The NETs contribute to the viscosity of the effusion, potentially contributing to its failure to clear as well as biofilm development. Our data indicates that Dornase alfa can fragment these strands and may play a role in future chronic OM treatment.

## Introduction

Recurrent acute otitis media (rAOM) affects between 10–20% of children [1], [2] and is a major reason for children requiring surgery, predominantly for ventilation tube insertion (VTI). Biofilm and intracellular infection have been demonstrated on the middle ear mucosa of children with chronic suppurative otitis media (CSOM), rAOM and chronic otitis media with effusion (OME) and are mechanisms of bacterial persistence in the middle ear causing recalcitrance to treatment and disease recurrence [3]–[6].

Biofilms on mucosal surfaces are a result of interactions between bacteria and the host and these differ physiologically from biofilms formed on inert surfaces [7]. DNA, both host and bacterially derived, is important in biofilm formation, stabilisation and persistence of nontypeable *Haemophilus influenzae* (NTHi) and *Streptococcus pneumoniae in vitro* and in the chinchilla model of otitis media (OM) [8]–[12]. DNA may be derived from active immune mechanisms such as neutrophil extracellular traps (NETs) [13], necrotic neutrophil presence [14], or produced directly by the bacteria [8]–[11]. NETs consist of a DNA backbone embedded with antimicrobial peptides and enzymes [15] and are important in confining infections and killing bacteria [16]. Some pathogens, including *S. pneumoniae* and NTHi can resist NET killing [9], [11], [15]–[17] and NTHi has been shown to actively elicit NET formation, using the DNA as a protective niche [18]. High levels of NETs may also lead to tissue damage as has been demonstrated in the liver and lung [19]. The presence of NETs and/or bacterial DNA in chronic OM may allow for novel treatments that breakdown DNA such as Dornase alfa used in the treatment of CF.

While middle ear mucosal biopsies have been used for demonstrating biofilm or intracellular presence of bacteria [3]–[6], biopsies are difficult to obtain and their small size limits analysis. The use of middle ear effusion (MEE) is an attractive alternative to examine the presence of biofilm-like formations in the middle ear and *Staphylococcus aureus* biofilm has been demonstrated in otorrhoea smears from children with CSOM [20]. Additional valuable data can be obtained from imaging the larger volume of MEE that cannot be acquired from single biopsies such as viability staining and fluorescent *in situ* hybridisation treatments, reducing bias in selecting probes and determining non-specific interactions between the probes and the sample. The presence of DNA in MEE from children with chronic and/or recurrent OM has not been previously demonstrated. In this study, we used FISH and viability staining to investigate whether bacterial biofilm, intracellular pathogens and extracellular DNA are present in MEE samples from children undergoing VTI for rAOM.

## Materials and Methods

### Patient population

MEE samples were obtained from children aged between 9 and 36 months with a history of at least 3 episodes of AOM and undergoing VTI as previously reported [21]. Children with diagnosed or suspected primary or secondary immunodeficiency, cystic fibrosis, immotile cilia syndrome, craniofacial abnormalities and chromosomal or genetic syndromes were excluded. Subjects were recruited from metropolitan hospitals in Perth, Western Australia, following written informed consent from parents or guardians. Approval for this study was obtained from the Princess Margaret Hospital for Children Ethics Committee (1295/EP) and from institutional review boards of hospitals where recruitment took place.

### Specimen acquisition and preparation

Samples were collected as previously described [21]. In brief, MEEs were aspirated through a myringotomy incision using a sterile Leukotrap® (Pall Corporation, New York, USA) connected to the surgical suction system. The tubing system was rinsed with sterile saline to recover all the MEE present into the Leukotrap®. Nasopharyngeal samples were collected using a sterile flexible cotton-wool tip swab (Copan, Brescia, Italy) and stored in STGGB.

### Bacterial culture

MEE (100uL) and nasopharyngeal samples (10uL) were assayed with standard culture techniques [22] on horse blood agar, chocolate agar containing bacitracin (300 mg/L), vancomycin (5 mg/L) and clindamycin (0.96 mg/L), and colistin nalidixic acid blood agar plates (Oxoid, Australia). Plates were incubated at 37°C in 5% CO_2_ for 48 h and all predominant bacteria recorded.

### Evaluation of bacterial viability and presence of DNA stranding in the MEE with BacLight staining kit

Viability of bacteria in the MEEs was assessed using the generic nucleic acid labelling of cells with the BacLight kit (Invitrogen Technologies). Briefly, 50ul of fresh MEE in saline was air dried for 30 min at room temperature on SuperFrost plus slides (Menzel-Glaser, Portsmouth, New Hampshire). Samples were incubated with 50 µl of the BacLight working solution (consisting of propidium iodide and SYTO9) (Invitrogen Technologies, Thornton, NSW) for 30 min at room temperature before three 5 min washes with PBS. Slides were mounted using an in-house low fade mounting media and stored at −20°C until CLSM imaging using the Leica TCS SP2 AOBS multiphoton confocal.

### Evaluation of MEE specimens using 16S rRNA FISH with pathogen-specific probes

FISH was conducted as described by Hall-Stoodley *et al* and Thornton *et al* [5], [6] for mucosal biopsies using 16S rRNA probes labelled with AlexaFluor 488, 546 or 633 dyes (Invitrogen Technologies, Thornton, NSW). A panel of probes was used to assess 5 slides for each MEE. Due to the availability of 4 lasers a maximum of 3 probes and one nucleic acid stain could be used on each sample. The probes included EUB338, a universal probe that hybridises all bacteria (with a 5′-3′ sequence, GCT GCC TCC CGT AGG AGT) [23], Spn probe (GTG ATG CAA GTG CAC CTT) for *S. pneumoniae* [24], Haeinf (CCG CAC TTT CAT CTT CCG) for *H. influenzae* [25], Sau (GAA GCA AGC TTC TCG TCC G) for *S. aureus* [24], Mrc88 (CCG CCA CUA AGU AUC AGA) for *Moraxella catarrhalis* [5] and Psae (TCT CGG CCT TGA AAC CCC) for *Pseudomonas aeruginosa* [26] and NONEUB338 as the negative control for non-specific hybridisations [27]. Specificity of 16S rRNA probes was extensively tested and validated using ATCC and clinical isolates.

MEE samples stored in 50% PBS/Ethanol at −20°C were used for FISH. A 50 µl aliquot from each MEE was air dried as above, incubated for 30 min at room temperature in 80% and then 100% ethanol. Samples were transferred to hybridisation chambers and incubated with lysozyme (10 mg/ml lysozyme in 0.1 M Tris (hydroxymethyl) aminomethane hydrochloride and 0.05 M Na_2_ EDTA) for 2 h at 37°C. Slides were then washed with PBS. Samples were treated with 40 µl of hybridisation buffer and 50 ng/ml of each probe at 46°C inside a hybridisation chamber for 2 h. Slides were then washed for 15 min at 48°C and incubated overnight with 1.2 µg/ml Hoechst 33342 (Invitrogen Technologies, Thornton, NSW) to stain the nuclei of the host cells. Slides were mounted in low fade mounting media and imaged using four colour CLSM using the Leica TCS SP2 AOBS multiphoton confocal. Images were analysed for presence of bacteria, specific bacterial species, biofilm structure and composition.

### Image analysis and interpretation

Samples were scored positive for biofilm presence when CLSM images showed bacterial aggregates resembling microcolonies or more extensive morphology, bacterial morphology based on size (0.5–2 µm) and shape (cocci or coccobacilli) was correct, biofilm ultrastructure was present and fluorescent signal was only present in the appropriate fluorescent channel. Host cells were identified through the use of the Hoechst 33342 nucleic acid stain. Bacteria were marked as being intracellular when discrete clusters of bacteria were in close proximity to intact host cell nuclei as per Thornton *et al* [6].

### Neutrophil elastase staining to determine whether the DNA in the samples was neutrophil derived

A subset of 10 MEEs was evaluated for neutrophil elastase by staining with anti-human neutrophil elastase antibodies (Abcam, UK). Fifty µl of MEE in saline was air dried for 30 min at room temperature on SuperFrost plus slides (Menzel-Glaser, Portsmouth, New Hampshire). MEE was blocked with Zuk's blocking buffer and incubated at room temperature for 30 min. Block buffer was removed and MEE was incubated with a 1/100 dilution of rabbit anti-neutrophil elastase (Abcam, UK) in Zuk's buffer with 1% saponin (Sigma, St Louis, MO) for 60 min at room temperature. Antibody was aspirated and the MEE was washed 3×10 min in PBS. MEE was then incubated with 1/100 AlexaFluor 647 labelled goat anti-rabbit antibody (Invitrogen Technologies, Thornton, NSW) in Zuk's buffer with 1% saponin for 60 min at room temperature in the dark. Antibody was aspirated and the MEE was washed 3×10 min in PBS at room temperature before being counterstained with propidium iodide (Invitrogen Technologies, Thornton, NSW), mounted in low fade mounting media and stored at −20°C until imaging using the Leica TCS SP2 AOBS multiphoton confocal.

### Evaluation of actin presence using Phalloidin staining to determine whether neutrophil DNA was released via an active mechanism or necrosis

Seven MEEs were evaluated for actin presence by staining with Phalloidin. Briefly, 50ul of MEE in saline was air dried for 30 min at room temperature on SuperFrost plus slides. Samples were incubated with 50 µl of TRITC labeled Phalloidin (Invitrogen Technologies, Thornton, NSW) at room temperature for 60 min, washed 3 times with PBS, stained for 30 min at room temperature with 50 µl of SYTO9 and washed 3×5 min in PBS. Slides were mounted using a low fade mounting media and stored at −20°C until imaging using the Leica TCS SP2 AOBS multiphoton confocal.

### Lectin staining to elucidate whether bacterial formations were surround by a matrix

A subset of 10 MEEs was stained for lectins by staining with Concanavalin A (ConA) and Wheat Germ Agglutinin (WGA). Fifty µl of MEE in saline was air dried for 30 min at room temperature on SuperFrost plus slides. MEE were fixed in 4% formaldehyde for 15 min at 37°C. Samples were then washed 3×10 min with PBS at room temperature. Samples were incubated at room temperature in the dark with 50 µl of a 5 µg/ml of WGA conjugated with Oregon Green 488 (Molecular Probes, Eugene, Oregon) in PBS for 10 min. Samples were washed twice in PBS. MEEs were then stained with 50 µl of 50 µg/ml FITC labelled ConA (Sigma, St Louis, MO) for 30 min at room temperature. Samples were then washed 3×5 min in PBS before being counterstained with propidium iodide and mounted in low fade mounting media and stored at −20°C until imaging.

### Dornase alfa treatment of middle ear fluid

Seven MEEs were treated with two concentrations of a highly purified solution of recombinant human deoxyribonuclease I (Dornase alfa, Pulmozyme, Roche) which is an enzyme that selectively cleaves DNA. Briefly, 250 µl MEE was incubated with 250 µl of 5 ug/ml or 1 mg/ml Dornase alfa for 15 min at room temperature. Samples were centrifuged using a serofuge and rinsed three times using PBS and stained as previously described using the BacLight kit (Invitrogen Technologies) followed by CLSM imaging.

## Results

### Patient characteristics

Thirty eight MEEs obtained from 24 children undergoing VTI for rAOM were assessed for the presence of extracellular DNA and live bacteria using generic nucleic acid staining and CLSM. Five children were female (20%) and the median age was 17.9 months (range 9.7–36.0 months; Table 1). All children enrolled in the study had received the 7-valent pneumococcal conjugate vaccine (Prevenar, Pfizer) at 2, 4 and 6 months of age as part of the Australian National Immunisation Program.

**Table 1:** Summary of demographic data and the pathogens identified in MEE from 24 children undergoing VTI for rAOM. Pathogens were identified using standard culture and FISH, and viability was determined using BacLight staining.

						FISH 16S RNA Probe									
Child No.	Sex (Age,m)	NP culture	Abx	Ear	MEE culture	EUB	Spn	Hi	Mcat	Sau	PA	BacLight (Live/Dead/Both)	Biofilm present	Intracellular bacteria observed	NETs
1	M (16.6)	Spn, Hi	Y	L	−	+	+	−	+	−	−	na	+	−	na
		Mcat		R	−	**+**	+	−	+	−	−	na	+	−	na
2	M (28.3)	Spn, Hi	N	L	−	**+**	−	−	−	−	−	Both	−	−	+
3	M (16.8)	Hi	Y	L	Sau	**+**	+	+	−	−	−	Live	+	−	+
				R	Sau	**+**	+	+	−	+	−	Live	+	+	+
4	M (20.0)	−	Y	R	−	**+**	−	−	−	−	−	Both	+	−	−
5	M (14.0)	−	Y	L	−	**+**	+	+	−	−	−	Both	+	+	−
				R	−	**+**	+	−	−	−	−	Live	+	−	+
6	M (23.3)	Hi	Y	L	Hi	**+**	−	+	−	+	−	Both	+	+	+
				R	Hi	**+**	+	+	−	−	−	Both	+	+	+
7	M (14.1)	Spn, Hi	N	R	Hi	**+**	+	+	−	+	−	Both	+	+	+
8	F (24.6)	Hi,	N	L	−	**+**	+	+	−	+	−	−	+	−	+
		Mcat		R	−	**+**	+	+	+	+	−	Both	+	+	+
9	F (18.0)	Spn	N	L	−	**+**	−	+	−	+	−	−	+	+	+
				R	−	**+**	+	−	+	−	−	Both	−	−	+
10	M (10.7)	Spn, Hi	N	R	Spn	**+**	+	+	−	−	−	Live	+	+	+
11	M (13.2)	Hi	N	L	Hi	**+**	−	+	−	−	−	Both	+	−	+
				R	Hi, Sau	**+**	−	+	−	−	−	Live	+	+	+
12	M (13.3)	Hi	N	R	−	**+**	+	−	+	−	−	Live	+	+	+
13	M (14.0)	Spn, Hi	Y	L	Spn, Hi	**+**	+	−	−	−	−	Live	+	−	+
				R	Spn, Hi	**+**	+	−	−	−	+	Live	−	−	+
14	F (26.6)	Hi,	U	L	−	**+**	+	+	−	−	−	Live	+	−	+
		Mcat		R	−	**+**	+	+	+	+	−	Live	+	−	+
15	M (17.9)	−	Y	L	−	**+**	+	+	+	−	−	Live	+	+	+
				R	AO	**+**	−	+	−	−	−	Live	+	+	+
16	M (18.5)	Sau	N	R	−	**+**	+	+	−	−	−	Live	+	+	−
17	F (16.9)	Spn, Hi	N	L	−	na	na	na	na	na	na	Live	+	+	+
		Mcat		R	−	na	na	na	na	na	na	Live	+	+	+
18	M (36.0)	−	Y	R	−	na	na	na	na	na	na	Both	+	−	+
19	M (9.7)	Mcat	N	R	−	na	na	na	na	na	na	Live	+	+	+
20	M (9.7)	Mcat, Hi	N	L	−	na	na	na	na	na	na	Live	+	−	+
				R	−	na	na	na	na	na	na	Live	+	−	+
21	M (16.5)	−	Y	L	−	na	na	na	na	na	na	Both	+	−	+
22	M (32.0)	−	N	L	−	na	na	na	na	na	na	Both	+	+	+
				R	−	na	na	na	na	na	na	Live	+	−	+
23	F (21.3)	Hi	U	L	−	na	na	na	na	na	na	Live	−	−	+
				R	−	na	na	na	na	na	na	−	−	−	+
24	M (18.7)	−	Y	L	−	na	na	na	na	na	na	−	−	−	+
					11/38	26/26	19/26	17/26	7/26	7/26	1/26	32/36	32/38	17/38	33/36
Number positive					(29%)	(100%)	(73%)	(65%)	(27%)	(27%)	(4%)	(89%)	(84%)	(45%)	(92%)

Biofilm and intracellular bacterial presence were also assessed.

Abbreviations: EUB – eubacterial probe, Spn – *S. pneumoniae* (organism and probe), Hi –*H. influenzae* (organism and probe), Mcat – *M. catarrhalis* (organism and probe), PA – *P. aeruginosa* (organism and probe), Sau – *S. aureus* (organism and probe), AO – *A. otitidis* (organism). na – Not available, Abx – taking antibiotics currently, U – Unknown, NP – nasopharyngeal culture.

### Culture of nasopharyngeal swabs and MEE

Eighteen children had at least one pathogen isolated from the nasopharynx, with 14 children carrying NTHi (58%), 6 *M. catarrhalis* (25%), 7 *S. pneumoniae* (29%) and 1 *S. aureus*. Nine children had 2 or more otopathogens isolated from the nasopharynx. Eleven MEEs (29%) were culture positive for at least one bacterial species, with 3 being positive for *S. pneumoniae* and 6 for NTHi (Table 1). Three MEEs were culture positive for *S. aureus* and 1 was culture positive for *Alloiococcus otitidis*. When bilateral effusions were collected, the same pathogen was isolated from both ears in 3/5 occasions (Table 1).

### Bacteria in MEE are viable and present in biofilm morphologies

**Figure 1:**
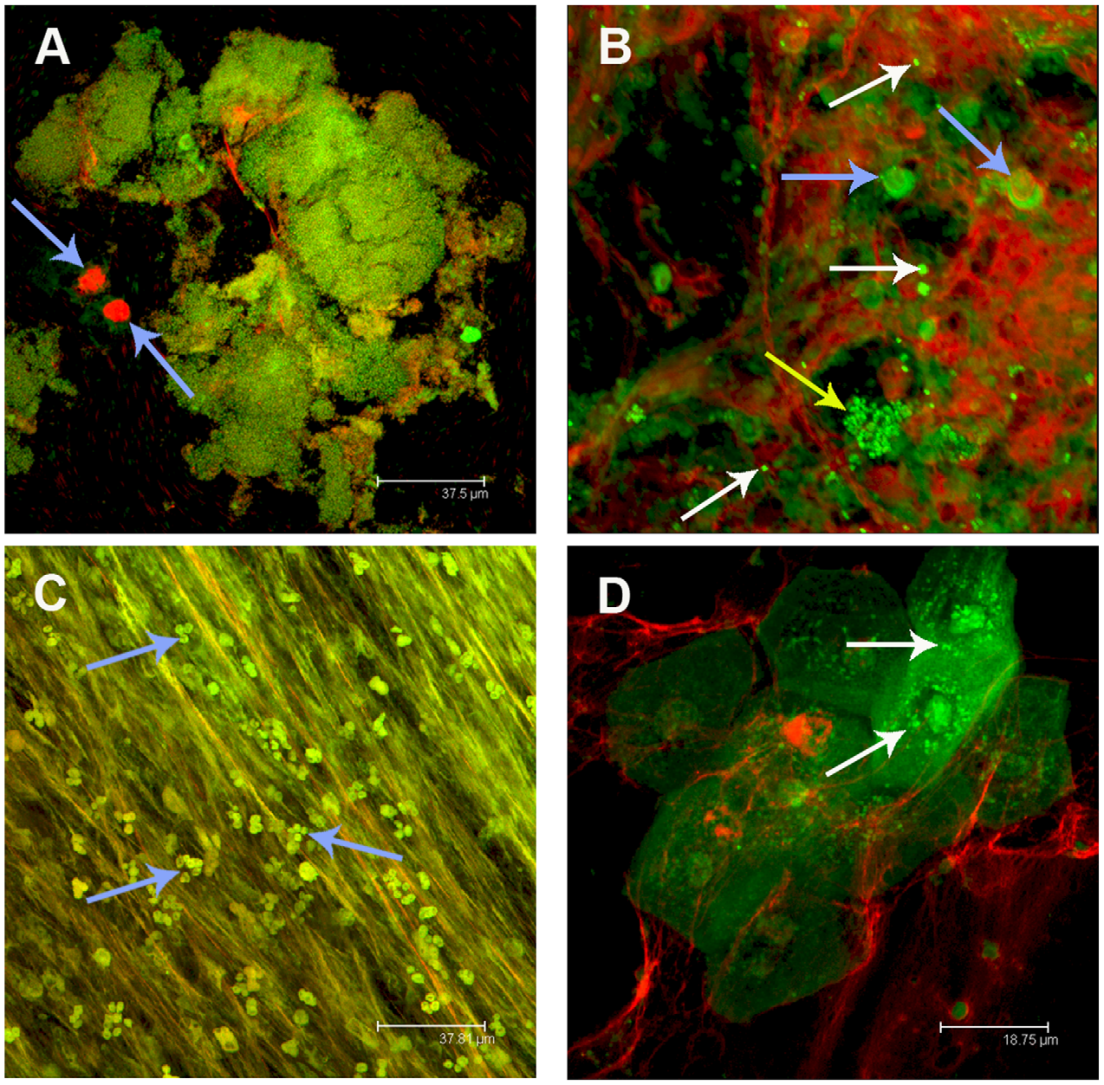
Biofilms (containing live, dead and intracellular bacteria) and DNA stranding were observed in MEE from children with rAOM. Representative images from MEEs stained with the BacLight kit. **A)** Large biofilm structures were observed. Both live (green) and dead bacteria (red) were present as were host cells (blue arrows). Bacteria with both dyes are evident (yellow). Host derived DNA strands were also present. **B)** Microcolonies of live bacteria (green; yellow arrow) within extensive nucleic acid matrices staining mainly with the propidium iodide component of the BacLight kit (red) were present throughout. Single bacteria (white arrows) and intact host nuclei were also observed (blue arrows). **C)** Extensive DNA stranding was present as were multi-lobed nuclei of intact neutrophils (blue arrows) apparent within the fibrotic DNA structure. **D)** Sloughed off epithelial cells containing live bacteria (arrows) were present in several MEEs.

**Figure 2:**
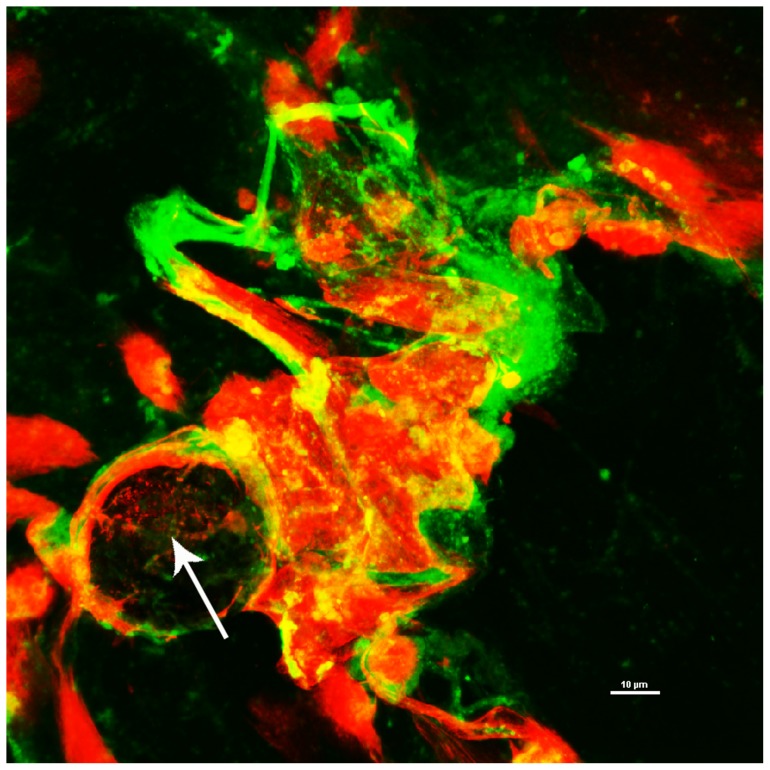
Bacterial biofilm matrices were demonstrated through staining with lectins. Concanavalin A (ConA) and Wheat Germ Agglutinin (WGA) (green) and propidium iodide (red) to stain for DNA. Co-localisation of the DNA and lectins are observed (yellow) suggesting that these are part of a biofilm structure. Bacterial biofilm can be observed (arrow) with bacteria being surrounding by a matrix that binds the lectin. Scale 10 µm.

To investigate whether bacteria within the MEE were viable despite low culture rates we used BacLight viability staining. Live bacteria were identified in 32 of the 36 MEE samples tested. In 20 of these MEE only live bacteria were present, while in 12 both live and dead bacteria were identified. Bacteria were present in extensive biofilms, microcolonies and in the planktonic state (Figure 1). Nucleic acid staining revealed extensive extracellular DNA stranding in 33 of the 36 MEE samples (Figure 1B and 1C). These extensive DNA structures frequently had biofilms associated. To assess the association of the bacteria with an extracellular matrix, MEEs were stained with lectins. The 2 lectins bound the glycoproteins in the biofilm matrix, and these were associated with both the bacteria and the DNA strands (Figure 2). This association suggests the DNA may be a structural component of the biofilms within the MEE.

### Polymicrobial biofilms containing known otopathogens were demonstrated in MEE

**Figure 3:**
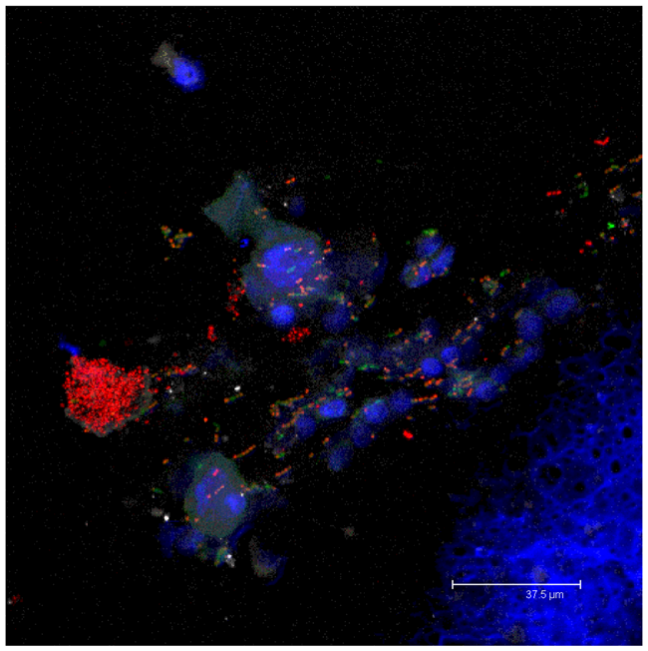
Multi-species bacterial biofilms containing known otopathogens, were demonstrated in the MEE of children with rAOM. Representative merged maximum intensity projection confocal image from FISH and Hoechst stained MEE (Child 3). MEE was culture positive for *S. aureus*. FISH: *S. pneumoniae* (AF488 green); universal EUB338 probe (AF546 red) *H. influenzae* (AF633 grey) Hoechst 33342 staining of host cell nuclei (blue). Chains of *S. pneumoniae* can be observed in this sample as well as those which are intracellular. Other unidentified bacteria (red) can be seen in chains and microcolony structures throughout this MEE.

We conducted species specific FISH on 26 MEEs. All 26 MEE samples were positive for bacteria when tested with the universal EUB338 probe. Two or more otopathogenic species were observed in 70% of effusions (Table 1 and Figure 3) showing the presence of polymicrobial biofilms in the middle ear. Nineteen of 26 (73%) samples were positive for *S. pneumoniae*, 17 (65%) for *H. influenzae*, 7 (27%) for *M. catarrhalis*, 7 (27%) for *S. aureus* and 1 (4%) was positive for *P. aeruginosa*. Using the NONEUB338 probe non-specific hybridisation was not observed for any of the MEE samples (data not shown). Bacteria were evident within epithelial cells (based on morphology – Figure 1D), though specific staining to identify these cells was not performed.

### Extensive DNA stranding evident within the MEE is largely NET derived

**Figure 4:**
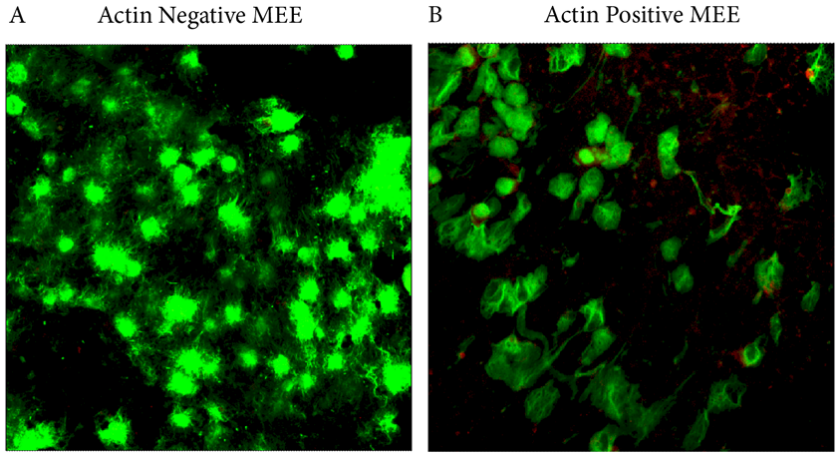
Most DNA present in the MEE was negative for actin, indicating it was actively produced and not released due to necrosis. MEE were stained with phalloidin to detect actin presence. MEEs were treated with the nucleic acid stain SYTO9 (green) and TRITC labelled Phalloidin (red). A) No red fluorescence was observed in most samples indicating actin was not present. This compared to B) where some actin is present (red). Even when the samples were positive for actin, this was not extensive, suggesting a combination of active DNA release and the presence of necrotic neutrophils is likely.

**Figure 5:**
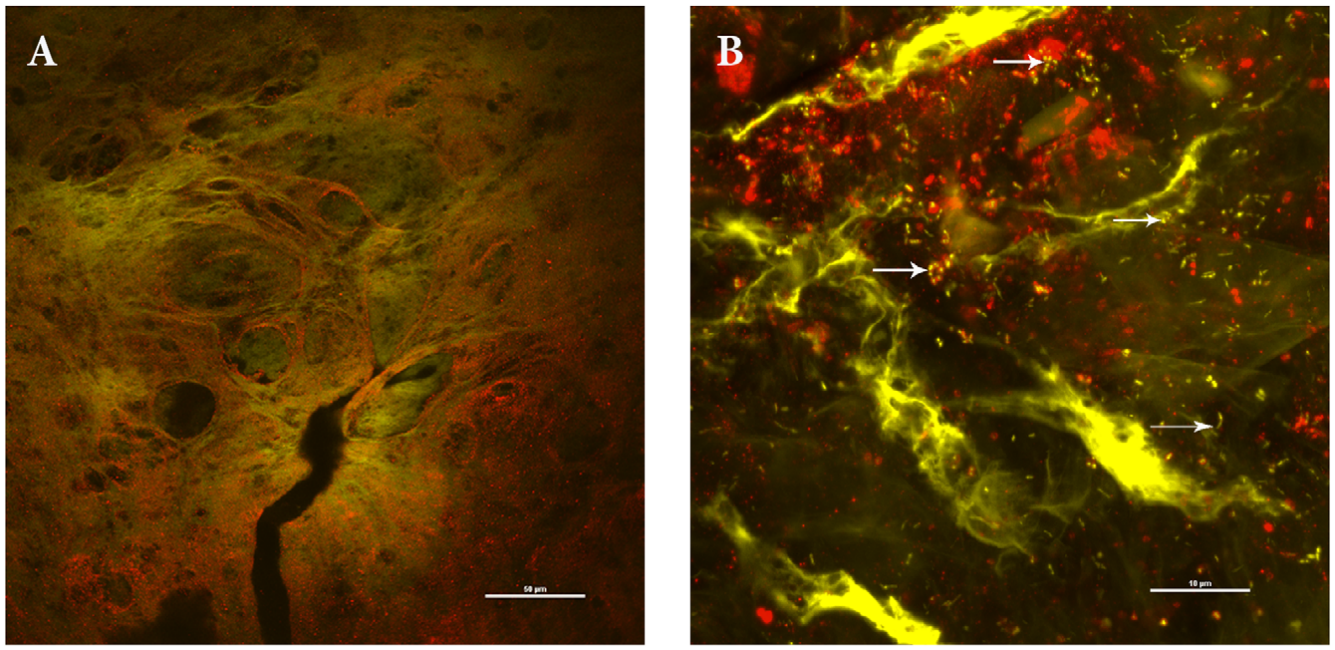
Most DNA present in the MEE was neutrophil-derived. MEE stained with neutrophil elastase AlexaFluor647 (Red) and propidium iodide (Yellow) and. A) Co-localisation of DNA and neutrophil elastase is present throughout the sample indicating the DNA that is present is neutrophil derived. Scale 50 µm. B) Co-localisation of DNA and neutrophil elastase is present throughout the sample as well as being bacterially associated throughout the sample (arrows). Scale 10 µm.

Given the extensive DNA stranding present and high proportion of neutrophil nuclei evident by morphology (Figure 1C) we investigated whether the DNA was derived from neutrophils. Cells were confirmed to be neutrophils in a selection of samples using haematoxylin and eosin staining, as well as CD15 staining (data not shown). Cells positive for the histiocytic lineage marker (CD68), which is indicative of macrophages were also observed (data not shown). Samples were stained with Phalloidin to determine if the cytoplasmic marker actin was present thereby implying that host derived DNA strands were due to degradation of necrotic neutrophils rather than produced as an active response by the neutrophils. Of the 7 samples tested only one was positive for actin (Figure 4) but this was not associated with DNA. Another marker of neutrophil DNA release is the association of the DNA with the enzyme neutrophil elastase. When labelled with anti-neutrophil elastase antibodies granular staining colocalised with DNA (Figure 5A) and bacteria were observed to be associated with DNA and neutrophil elastase (Figure 5B).

### Treatment with Dornase alfa effectively breaks down the DNA structures and associated biofilms in the MEE

**Figure 6:**
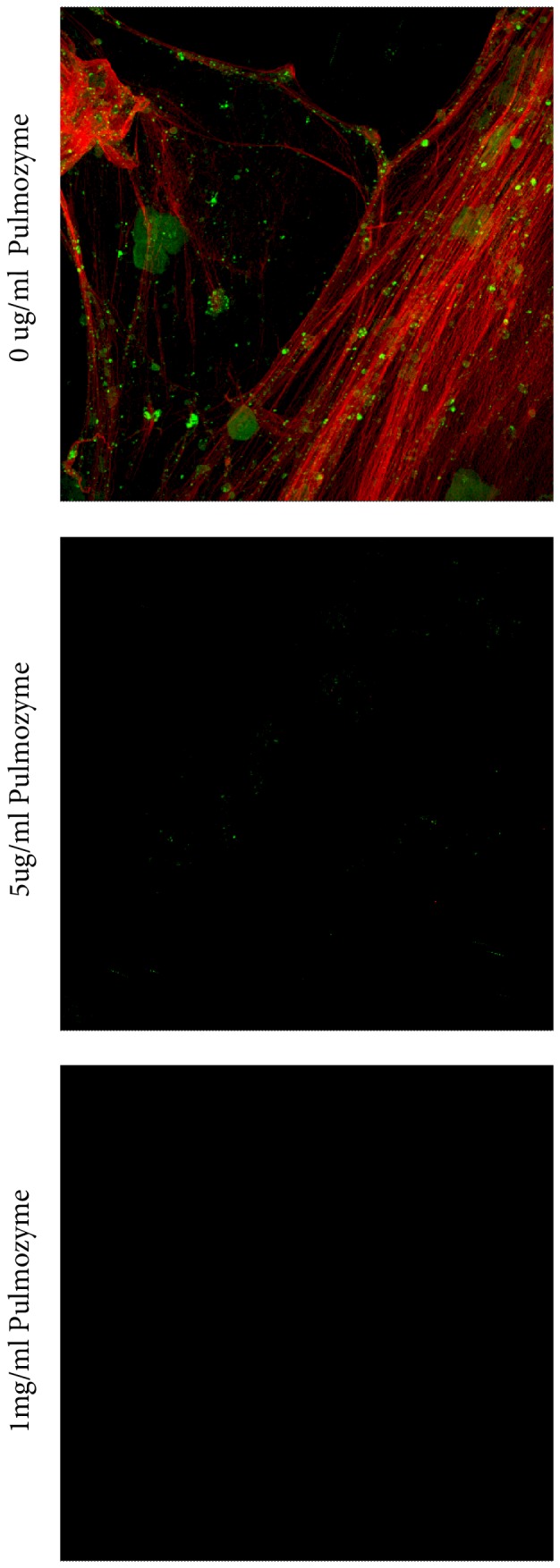
Dornase alfa (Pulmozyme®) treatment of MEE results in disintegration of the NET structures. DNA staining of MEEs from children with rAOM treated with Dornase alfa or left untreated. In the top panel, a control MEE untreated with Dornase alfa where extensive DNA stranding, epithelial cells and bacteria are evident. When treated with Dornase alfa even at concentrations as low as 5ug/ml complete fragmentation of the strands in the MEE are seen and nothing remains on the slide at 1 mg/ml.

When MEEs were treated with Dornase alfa at a concentration used in CF therapy (1 mg/ml) and at 5µg/ml, complete fragmentation of the DNA matrix was observed in all samples within 15 minutes (Figure 6).

## Discussion

Bacterial biofilm is increasingly recognised to play a role in the recurrence and persistence of infections such as OM. Demonstrating biofilm in very small tissue biopsies from the middle ear of children is technically difficult and may be biased by sampling and probe selection [5], [6]. This is the first study to identify multi-species biofilms consisting of live otopathogens in MEE samples from children with rAOM. Furthermore this is the first study to demonstrate the association of these biofilms with a DNA matrix produced by NETs which may represent a treatment target through the use of the therapeutic agent Dornase alfa which was able to fragment the DNA matrix.

In this study, bacterial biofilms were demonstrated in 84% of MEE from children undergoing VTI for rAOM. This is similar to rates of biofilm presence on the middle ear mucosa of children with chronic OME and rAOM (64–92%) [5], [6] and in CSOM discharge samples (83%) [20]. MEE biofilms appear similar to those described in bronchoalveoloar lavage fluids from children with CF, where bacteria adhere indirectly to the mucosa [28] resulting in small bacterial aggregates in highly viscous fluid [29]. Biofilms in the MEE were not homogenous, suggesting discrete microcolonies, similar to patchy biofilm formations on the middle ear mucosa in the chinchilla model of OM [30].

Using FISH, biofilms within MEE were shown to be polymicrobial, consistent with those observed in the environment [31] and previously identified on the middle ear mucosa [5], [6]. This is clinically relevant as in animal models, polymicrobial biofilms provide a protective advantage for bacteria compared to a single species biofilm [32]. Host inflammatory responses are increased when multiple bacterial species are present regardless of whether they are able to be cultured [33], [34]. While 92% of MEE's were positive for at least one of the known otopathogens, none bound the NONEUB338 (the nonsense or negative control) probe indicating the specificity of this technique. Large numbers of unidentified species were also present within the MEE (as identified by binding the EUB338 (universal) probe) and the high rates of bacterial detection using FISH suggest that this technique represents a sensitive and specific tool to assess bacteria present in MEE.

We demonstrated extensive extracellular DNA stranding in MEE, predominantly neutrophil derived through NET formation, and live bacteria within these. Both animal [10] and *in vitro* studies [8] suggest that the presence of such DNA, both host and bacterially derived, play a role in biofilm development and bacterial persistence in OM. NETs also play roles in interspecies competition for colonising the nasopharynx [35], [36] and may also do so in the middle ear. Similarly to previous studies [21], [37] we found *S. pneumoniae* and *H. influenzae* the most commonly identified pathogens in the MEE of children with rAOM. These species can resist NET killing despite high levels of antimicrobial proteins [9], [16], [18] due to production of DNases and through alteration of the bacterial surface [18]. The lipooligosaccharide modifications promoting NTHi survival in NETs are similar to those that promote biofilm formation [18]. In the chinchilla model of NTHi OM, a bacterial histone-like protein (IHF) is involved in structuring the extracellular DNA that plays a major role in stabilising biofilms [38]. The inability of NETs to clear otopathogens may contribute to establishing stable biofilm communities in the MEE [9], [11], [18]. This combined with the viscosity of the DNA may impede clearance of the MEE and the associated bacteria. Mast cells and eosinophils are also able to form extracellular traps [39] and their role in rAOM requires further investigation.

In the MEE, it is important to use both the nucleic acid staining and the specific FISH methodology to assess biofilms. Generic staining protocols such as the BacLight kit do not involve enzyme treatments or stringent wash steps, which are necessary to conduct FISH staining, which means that the biofilm morphology within the MEE is retained more reliably. Indeed biofilm presence was more often scored using this technique compared with using FISH. FISH however allows identification of specific species and their relationship within the sample. Intracellular pathogens were detected in 45% of effusions, and 36% of children. These rates are lower than those detected in biopsies from children in previous studies (71%) [6]. The lower detection rate likely reflects a higher proportion of cells containing pathogenic bacteria being sequestered within the tissue rather than shed into the MEE. While MEE may be useful to assess biofilm presence in the middle ear, it may not be as representative in determining intracellular sequestration.

The presence of non-cultureable but metabolically active bacteria in the MEE provides further evidence of biofilm involvement in OM. In this study 21% of children were culture positive for any of the main OM pathogens which is comparable with other published studies [5], [40]. However, using viability staining, 89% of MEEs tested were positive for live bacteria, predominantly *H. influenzae* followed by *S. pneumoniae*. Discrepancies were present comparing FISH and culture, with bacteria observed more often using direct microscopic examination. These differences have been observed previously when comparing standard culture and a direct detection method such as FISH [41], [42]. Detection of bacterial species in one ear of a single subject did not always correlate with the opposite ear using either culture or FISH. Findings of different pathogens in the ears of individual children are not isolated to our study [5] and may suggest the presence of different microbial reservoirs in each ear.

FISH represents a specific and sensitive tool for assessing bacteria in the MEEs and in addition provides spatial and morphological data. This could be important when considering larger scale studies, particularly monitoring clinical interventions to treat biofilm, where it is easier to collect and examine MEEs than biopsies. Using MEE to monitor intracellular persistence may not be as reliable as much lower rates of intracellular infection were observed in the MEE when compared to mucosal biopsies. Direct comparisons of MEE and middle ear biopsies from the same child are required to confirm this. Data from animal models have also demonstrated a difference in the niches occupied by bacterial species in polymicrobial infections. Specifically, in the chinchilla model of *H. influenzae* and *M. catarrhalis* OM, where *M. catarrhalis* is seen to be exclusively associated with the epithelial surface while *H. influenzae* is detected in both the epithelium and the MEE [32].

Studies often use nasopharyngeal carriage of otopathogens as a surrogate for the infecting pathogens of the middle ear. This may be indicative with acute infection but less reliable in children with recurrent AOM. In this small study, we did not see a direct correlation between the pathogens in the middle ear with any of the detection methodologies with those cultured from the nasopharynx. This suggests that different bacteria may be found only in the middle ear space reflecting the presence of biofilm as a separate source of microbes causing the recurrent acute infections. Investigation of MEE and NPS samples using molecular detection techniques rather than relying solely on culture methodology is required as previous studies have demonstrated that non-cultureable organisms can be present in up to 36% of NPS using molecular methods [43].

Dornase alfa is the only licensed DNAse with efficacy in the treatment of CF [44] and has been demonstrated to improve lung function, reduce DNA levels, and to reduce the viscosity of airway secretions [45]. When we treated MEEs with Dornase alfa in the current study, complete and rapid degradation of the matrix was observed even at concentrations lower than those used clinically in CF. While the number of MEEs tested was small, this data suggests that Dornase alfa may be a useful adjunct treatment in recurrent or chronic OM. Directly degrading the DNA may improve bacterial clearance from the middle ear by reducing biofilm stability and causing bacteria to return to their planktonic form which are susceptible to locally (or systemically) administered antimicrobials and host immune mechanisms. In *in vitro* systems of neutrophil induced biofilms by *P. aeruginosa*, DNases had decreased activity in treating thicker, more mature biofilms or those containing mucoid strains of bacteria, possibly due to increased polysaccharide masking DNA cleavage sites [29]. This effect of DNase appears to be species specific, [8], [12], [38] and needs to be better characterized for otopathogens and for polymicrobial biofilms. Other biofilm components in the MEE also need to be better assessed as they may affect the efficacy of a DNase treatment.

This study includes a relatively small number of children with a specific clinical presentation of OM, being persistent effusion following rAOM episodes. Investigation of the role of biofilm in MEE and the middle ear mucosa in a population of children with a broader OM spectrum, including children with chronic OME and CSOM, and the potential of Dornase alfa are required.

These data provide an insight into the role of biofilm in the middle ear which is not only surface associated but present in the entire middle ear cavity. Live bacteria in microcolonies are present in extensive DNA stranding from NETs in the MEE of children with rAOM, contributing to effusion viscosity and may affect its failure to clear. The involvement of these biofilms with NET associated DNA indicates a potential for the novel use of Dornase alfa combined with currently used topical antibiotics at the time of surgery. We are currently conducting a clinical trial to assess the use of Dornase alfa as an adjunct therapy to VTI to prevent recurrent episodes of OM and possibly the need for repeat surgery. The potential use of Dornase alfa as a treatment for recurrent or chronic OM prior to surgery requires development of suitable methods of delivery to the middle ear and this warrants further investigation.

## References

[1] Casselbrant ML, Mandel E (2003) Epidemiology. In: Rosenfeld RMB, C., editor. Evidence-Based Otitis Media. Second Edition ed. Hamilton, London: BC Decker Inc.

[2] Rosenfeld RM (2003) Clinical Pathway for Acute Otitis Media. In: Rosenfeld RM, & Bluestone C, editors. Evidence-Based Otitis Media. Hamilton, London: BC Decker Inc.

[3] Coates H (2004) Chronic suppurative otitis media without cholesteatoma. In: Alper C, Bluestone, C, Dohar, J, Madel, E & Casselbrant, M., editor. Advanced Therapy of Otitis Media. Hamilton, Ontario: B.C. Decker Incorporated. 299–305.

[4] Coates H, Thornton R, Langlands J, Filion P, Keil AD, et al (2008) The role of chronic infection in children with otitis media with effusion: evidence for intracellular persistence of bacteria. Otolaryngol Head Neck Surg 138: 778–781. 18503854 10.1016/j.otohns.2007.02.009

[5] Hall-Stoodley L, Hu FZ, Gieseke A, Nistico L, Nguyen D, et al (2006) Direct detection of bacterial biofilms on the middle-ear mucosa of children with chronic otitis media. Jama 296: 202–211. 16835426 10.1001/jama.296.2.202 PMC1885379

[6] Thornton RB, Rigby PJ, Wiertsema SP, Filion P, Langlands J, et al (2011) Multi-species bacterial biofilm and intracellular infection in otitis media. BMC Pediatr 11: 94. 22018357 10.1186/1471-2431-11-94 PMC3224757

[7] Post JC, Stoodley P, Hall-Stoodley L, Ehrlich GD (2004) The role of biofilms in otolaryngologic infections. Curr Opin Otolaryngol Head Neck Surg 12: 185–190. 15167027 10.1097/01.moo.0000124936.46948.6a

[8] Hall-Stoodley L, Nistico L, Sambanthamoorthy K, Dice B, Nguyen D, et al (2008) Characterization of biofilm matrix, degradation by DNase treatment and evidence of capsule downregulation in Streptococcus pneumoniae clinical isolates. BMC Microbiol 8: 173. 18842140 10.1186/1471-2180-8-173 PMC2600794

[9] Hong W, Juneau RA, Pang B, Swords WE (2009) Survival of bacterial biofilms within neutrophil extracellular traps promotes nontypeable Haemophilus influenzae persistence in the chinchilla model for otitis media. J Innate Immun 1: 215–224. 20375579 10.1159/000205937 PMC6951045

[10] Jurcisek JA, Bakaletz LO (2007) Biofilms formed by nontypeable Haemophilus influenzae in vivo contain both double-stranded DNA and type IV pilin protein. J Bacteriol 189: 3868–3875. 17322318 10.1128/JB.01935-06 PMC1913342

[11] Reid SD, Hong W, Dew KE, Winn DR, Pang B, et al.. (2009) Streptococcus pneumoniae Forms Surface-Attached Communities in the Middle Ear of Experimentally Infected Chinchillas. J Infect Dis. 10.1086/597042 19434911

[12] Moscoso M, Garcia E, Lopez R (2006) Biofilm formation by Streptococcus pneumoniae: role of choline, extracellular DNA, and capsular polysaccharide in microbial accretion. J Bacteriol 188: 7785–7795. 16936041 10.1128/JB.00673-06 PMC1636320

[13] Remijsen Q, Kuijpers TW, Wirawan E, Lippens S, Vandenabeele P, et al (2011) Dying for a cause: NETosis, mechanisms behind an antimicrobial cell death modality. Cell Death Differ 18: 581–588. 21293492 10.1038/cdd.2011.1 PMC3131909

[14] Walker TS, Tomlin KL, Worthen GS, Poch KR, Lieber JG, et al (2005) Enhanced Pseudomonas aeruginosa biofilm development mediated by human neutrophils. Infect Immun 73: 3693–3701. 15908399 10.1128/IAI.73.6.3693-3701.2005 PMC1111839

[15] Wartha F, Beiter K, Normark S, Henriques-Normark B (2007) Neutrophil extracellular traps: casting the NET over pathogenesis. Curr Opin Microbiol 10: 52–56. 17208512 10.1016/j.mib.2006.12.005

[16] Beiter K, Wartha F, Albiger B, Normark S, Zychlinsky A, et al (2006) An endonuclease allows Streptococcus pneumoniae to escape from neutrophil extracellular traps. Curr Biol 16: 401–407. 16488875 10.1016/j.cub.2006.01.056

[17] Brinkmann V, Zychlinsky A (2007) Beneficial suicide: why neutrophils die to make NETs. Nat Rev Microbiol 5: 577–582. 17632569 10.1038/nrmicro1710

[18] Juneau RA, Pang B, Weimer KE, Armbruster CE, Swords WE (2011) Nontypeable Haemophilus influenzae initiates formation of neutrophil extracellular traps. Infect Immun 79: 431–438. 20956567 10.1128/IAI.00660-10 PMC3019868

[19] Logters T, Margraf S, Altrichter J, Cinatl J, Mitzner S, et al (2009) The clinical value of neutrophil extracellular traps. Med Microbiol Immunol 198: 211–219. 19653000 10.1007/s00430-009-0121-x

[20] Homoe P, Bjarnsholt T, Wessman M, Sorensen HC, Johansen HK (2009) Morphological evidence of biofilm formation in Greenlanders with chronic suppurative otitis media. Eur Arch Otorhinolaryngol. 10.1007/s00405-009-0940-9 19283404

[21] Wiertsema SP, Kirkham LA, Corscadden KJ, Mowe EN, Bowman JM, et al.. (2011) Predominance of nontypeable Haemophilus influenzae in children with otitis media following introduction of a 3+0 pneumococcal conjugate vaccine schedule. Vaccine. 10.1016/j.vaccine.2011.05.035 21621576

[22] Watson K, Carville K, Bowman J, Jacoby P, Riley TV, et al (2006) Upper respiratory tract bacterial carriage in Aboriginal and non-Aboriginal children in a semi-arid area of Western Australia. Pediatr Infect Dis J 25: 782–790. 16940834 10.1097/01.inf.0000232705.49634.68

[23] Amann RI, Krumholz L, Stahl DA (1990) Fluorescent-oligonucleotide probing of whole cells for determinative, phylogenetic, and environmental studies in microbiology. J Bacteriol 172: 762–770. 1688842 10.1128/jb.172.2.762-770.1990 PMC208504

[24] Kempf VA, Trebesius K, Autenrieth IB (2000) Fluorescent In situ hybridization allows rapid identification of microorganisms in blood cultures. J Clin Microbiol 38: 830–838. 10655393 10.1128/jcm.38.2.830-838.2000 PMC86216

[25] Hogardt M, Trebesius K, Geiger AM, Hornef M, Rosenecker J, et al (2000) Specific and rapid detection by fluorescent in situ hybridization of bacteria in clinical samples obtained from cystic fibrosis patients. J Clin Microbiol 38: 818–825. 10655391 10.1128/jcm.38.2.818-825.2000 PMC86213

[26] Trebesius K, Leitritz L, Adler K, Schubert S, Autenrieth IB, et al (2000) Culture independent and rapid identification of bacterial pathogens in necrotising fasciitis and streptococcal toxic shock syndrome by fluorescence in situ hybridisation. Med Microbiol Immunol 188: 169–175. 10917153 10.1007/s004300000035

[27] Manz W, Amann R, Ludwig W (1992) Phylogenetic Oligodeoxynucleotide Probes for the Major Subclasses of Proteobacteria: Problems and Solutions. Systematic and Applied Microbiology: 593–600.

[28] Kobayashi H, Kobayashi O, Kawai S (2009) Pathogenesis and clinical manifestations of chronic colonization by Pseudomonas aeruginosa and its biofilms in the airway tract. J Infect Chemother 15: 125–142. 19554398 10.1007/s10156-008-0691-3

[29] Parks QM, Young RL, Poch KR, Malcolm KC, Vasil ML, et al (2009) Neutrophil enhancement of Pseudomonas aeruginosa biofilm development: human F-actin and DNA as targets for therapy. J Med Microbiol 58: 492–502. 19273646 10.1099/jmm.0.005728-0 PMC2677169

[30] Ehrlich GD, Veeh R, Wang X, Costerton JW, Hayes JD, et al (2002) Mucosal biofilm formation on middle-ear mucosa in the chinchilla model of otitis media. Jama 287: 1710–1715. 11926896 10.1001/jama.287.13.1710

[31] Stoodley P, Sauer K, Davies DG, Costerton JW (2002) Biofilms as complex differentiated communities. Annu Rev Microbiol 56: 187–209. 12142477 10.1146/annurev.micro.56.012302.160705

[32] Armbruster CE, Hong W, Pang B, Weimer KE, Juneau RA, et al.. (2010) Indirect Pathogenicity of Haemophilus influenzae and Moraxella catarrhalis in Polymicrobial Otitis Media Occurs via Interspecies Quorum Signaling. MBio 1. 10.1128/mBio.00102-10 PMC2925075 20802829

[33] Krishnamurthy A, McGrath J, Cripps AW, Kyd JM (2009) The incidence of Streptococcus pneumoniae otitis media is affected by the polymicrobial environment particularly Moraxella catarrhalis in a mouse nasal colonisation model. Microbes Infect 11: 545–553. 19306940 10.1016/j.micinf.2009.03.001

[34] Ratner AJ, Lysenko ES, Paul MN, Weiser JN (2005) Synergistic proinflammatory responses induced by polymicrobial colonization of epithelial surfaces. Proc Natl Acad Sci U S A 102: 3429–3434. 15728393 10.1073/pnas.0500599102 PMC552945

[35] Lysenko ES, Ratner AJ, Nelson AL, Weiser JN (2005) The role of innate immune responses in the outcome of interspecies competition for colonization of mucosal surfaces. PLoS Pathog 1: e1. 16201010 10.1371/journal.ppat.0010001 PMC1238736

[36] Pettigrew MM, Gent JF, Revai K, Patel JA, Chonmaitree T (2008) Microbial interactions during upper respiratory tract infections. Emerg Infect Dis 14: 1584–1591. 18826823 10.3201/eid1410.080119 PMC2609881

[37] Dagan R, Leibovitz E, Greenberg D, Yagupsky P, Fliss DM, et al (1998) Early eradication of pathogens from middle ear fluid during antibiotic treatment of acute otitis media is associated with improved clinical outcome. Pediatr Infect Dis J 17: 776–782. 9779760 10.1097/00006454-199809000-00005

[38] Goodman SD, Obergfell KP, Jurcisek JA, Novotny LA, Downey JS, et al.. (2011) Biofilms can be dispersed by focusing the immune system on a common family of bacterial nucleoid-associated proteins. Mucosal Immunol. 10.1038/mi.2011.27 21716265

[39] Papayannopoulos V, Metzler KD, Hakkim A, Zychlinsky A (2010) Neutrophil elastase and myeloperoxidase regulate the formation of neutrophil extracellular traps. J Cell Biol 191: 677–691. 20974816 10.1083/jcb.201006052 PMC3003309

[40] Gok U, Bulut Y, Keles E, Yalcin S, Doymaz MZ (2001) Bacteriological and PCR analysis of clinical material aspirated from otitis media with effusions. Int J Pediatr Otorhinolaryngol 60: 49–54. 11434953 10.1016/s0165-5876(01)00510-9

[41] Heiniger N, Spaniol V, Troller R, Vischer M, Aebi C (2007) A reservoir of Moraxella catarrhalis in human pharyngeal lymphoid tissue. J Infect Dis 196: 1080–1087. 17763332 10.1086/521194

[42] Kirketerp-Moller K, Jensen PO, Fazli M, Madsen KG, Pedersen J, et al (2008) Distribution, organization, and ecology of bacteria in chronic wounds. J Clin Microbiol 46: 2717–2722. 18508940 10.1128/JCM.00501-08 PMC2519454

[43] Smith-Vaughan H, Byun R, Nadkarni M, Jacques NA, Hunter N, et al (2006) Measuring nasal bacterial load and its association with otitis media. BMC Ear Nose Throat Disord 6: 10. 16686940 10.1186/1472-6815-6-10 PMC1479363

[44] Henke MO, Ratjen F (2007) Mucolytics in cystic fibrosis. Paediatr Respir Rev 8: 24–29. 17419975 10.1016/j.prrv.2007.02.009

[45] Ratjen F, Paul K, van Koningsbruggen S, Breitenstein S, Rietschel E, et al (2005) DNA concentrations in BAL fluid of cystic fibrosis patients with early lung disease: influence of treatment with dornase alpha. Pediatr Pulmonol 39: 1–4. 15532079 10.1002/ppul.20134

